# How effective are protected areas for reducing threats to biodiversity? A systematic review protocol

**DOI:** 10.1186/s13750-023-00311-4

**Published:** 2023-09-08

**Authors:** Katherine Pulido-Chadid, Elina Virtanen, Jonas Geldmann

**Affiliations:** 1 Center for Macroecology, Evolution and Climate, Globe Institute, University of Copenhagen, Copenhagen, Denmark; 2 Finnish Natural History Museum, University of Helsinki, Helsinki, Finland; 3 Finnish Environment Institute, Helsinki, Finland

**Keywords:** Protected areas, Conservation, Threats to biodiversity, Protected areas effectiveness, Threat reduction in protected areas

## Abstract

**Background:**

Protected areas (PAs) have become one of the most important instruments to preserve nature and, when effective, can significantly reduce human pressure and derived threats to biodiversity. However, evidence suggests that despite the growing number and coverage of PAs worldwide, biodiversity trends continue to deteriorate, and human pressure increases outside and inside PAs. While many studies have focused on the effectiveness of PAs in maintaining ecological features, less attention has been given to the threat reduction potential of PAs, despite threats being one of the main factors leading to the need to conserve biodiversity. It is therefore essential to understand PAs' role in addressing threats. In this paper, we describe the protocol for conducting a systematic review to explore and review the evidence surrounding the effectiveness of PAs as an intervention to reduce threats to biodiversity. We will examine the role of PAs in addressing several types of threats. Thus, our primary research question is: How effective are protected areas for reducing threats to biodiversity?

**Methods:**

This protocol follows the Collaboration for Environmental Evidence guidelines for evidence synthesis and complies with the ROSES (Reporting Standards for Systematic Evidence Synthesis) reporting framework. We will use a comprehensive search, covering databases such as Web of Science—core collection and Scopus and organizational websites to capture relevant grey literature. Our search terms and strategies aim to find studies assessing change of threats given in PAs at any scale and ecosystem type capturing literature in English. Independent reviewers will screen search results at the title—abstract, and full text levels. In order to evaluate the relevance of the evidence, we will use the Collaboration for Environmental Evidence Critical Appraisal Tool. The results will be presented as a narrative synthesis supported by quantitative data. Additionally, a meta-analysis, if possible, will be performed.

**Supplementary Information:**

The online version contains supplementary material available at 10.1186/s13750-023-00311-4.

## Background

In recent decades, a biodiversity crisis unseen in human history has been ongoing, with the current rate of species extinctions greatly exceeding the estimated background rate [1, 2]. The current pace and rates of biodiversity loss have been attributed to the pressure inflicted on natural systems due to human activities, which threaten biodiversity [3]. According to the IUCN, threats to biodiversity are: “The proximate human activities or processes that have caused, are causing, or may cause the destruction, degradation, and/or impairment of biodiversity targets” [4]. Among such activities, direct threats are those that have immediate and evident impacts, including unsustainable fishing or hunting practices, oil drilling, pollution, and road construction, among others [5]. At the global level, the major drivers of biodiversity loss are land use change, resource extraction, pollution, invasive alien species, and climate change. Those five threats are driven by the different societal values and behaviors that emerge from different regions, such as production and consumption patterns, human population dynamics, trade, technological innovations, and governance [6].

Approximately 75% of the earth's surface has been altered by human activities, which is strongly associated with biodiversity loss [6]. As a measure to limit human activities and provide a safe space for nature to thrive, protected areas (PAs) have become one of the most important instruments of nature conservation and, when effective, can significantly reduce human pressure and derived threats to biodiversity [7–9]. In order to halt global biodiversity loss, worldwide agreements have been seeking to protect biodiversity by increasing the coverage and management effectiveness of PAs [10]. As a result, by 2021, PAs covered 16.6% of terrestrial and freshwater ecosystems and 7.7% of marine ecosystems [11]. With further aims to achieve global effective conservation and management of at least 30% of the land, freshwater, coastal and marine ecosystems by 2030, as recently agreed at the 15th COP of the CBD [12]. However, the evidence suggests that despite the growing number and coverage of PAs worldwide, biodiversity trends continue to deteriorate [13, 14], while human pressure increases outside and inside PAs, especially in the tropics [15, 16]. It has therefore proved ineffective to merely expand PA cover to achieve conservation success [17].

Aligned with the 2030 Agenda for Sustainable Development, the Kunming-Montreal Global Biodiversity Framework establishes a comprehensive plan to transform the relationship between societies and biodiversity by 2030. The Framework recognizes the impact of human activities and the consequent threats posed to nature. Goal A of the Framework seeks to preserve, enhance, or restore all ecosystems' integrity, connectivity, and resilience through sustainable conservation practices. By 2050, the aim is to increase the area of natural ecosystems, prevent the human-induced extinction of known threatened species, reduce the extinction rate and risk of all species, and increase the abundance of native wild species. To achieve these goals, the Framework includes eight targets to reduce the five main threats to biodiversity and improve the effective conservation and management of PAs and other effective area-based conservation measures [12]. By implementing measures to reduce threats, the Framework seeks to promote the long-term conservation and sustainable use of biodiversity, thus supporting achieving its broader goals. Improving the quantity and quality of PAs is essential for achieving sustainable development and restoring biodiversity. However, to succeed, it is necessary to better understand the role of interventions within PAs in addressing threats. Gathering exhaustive evidence on how PAs and their management effectiveness have affected the occurrence and intensity of threats to biodiversity is crucial to achieving these goals.

The effectiveness of PAs is a function of a variety of factors, including both decisions made at the time of establishment (extent, design, location, connectivity, representativeness) and subsequent management decisions [18]. According to the IUCN, an effective PA is: “*a clearly defined geographical space, recognized, dedicated and managed, through legal or other effective means, to achieve the long-term conservation of nature with associated ecosystem services and cultural values*” [19]. The concept of PAs effectiveness is broad as well as the means of measurement, thus effectiveness has been assessed by examining their extension, coverage of species and ecosystems, their management effectiveness, social, cultural and biodiversity outcomes, among other attributes [18, 20, 21]. A challenging aspect of PAs performance assessment is evaluating the relative impact of driving factors since PAs are embedded within complex social and ecological systems [22]. The current study will examine the ecological dimensions of effectiveness that are a direct indicator of threat abatement.

The ecological effectiveness of PAs can be determined by their representation and persistence of ecological features, such as biodiversity attributes, ecosystem services, and ecological processes, which the sites or networks of sites aim to protect [22, 23]. The representation of a PA refers to the scope, number, and extent of these features present in the area while persistence is determined by how well they are protected against existing pressures and how they change over time [22]. Simultaneously, interlinked factors such as location, spatial design, management, and threats directly affect the ecological effectiveness of PAs [18, 22]. Overall, the extent to which PAs can effectively conserve nature over the long term is determined by how well they abate threats through effective management and enhance resilience through location and design factors [18, 24, 25]. Consequently, management and threat reduction strategies are vital for assessing effectiveness [26].

Numerous studies have analyzed the impact of PAs by considering different factors related to PAs effectiveness. A systematic review by Geldmann et al. [23] has documented the ability of PAs to reduce rates of biodiversity loss compared to unprotected sites [23], but the effectiveness varies due to the socio-economic context and management conditions [27, 28]. For instance, PAs in regions with higher development indicators show a positive association with wildlife populations [29], highlighting that management and socio-economic conditions are crucial to achieving the targets of biodiversity protection and threat reduction [27].

Other studies have focused on the potential of PAs in reducing habitat loss [15, 23, 30] and species population dynamics [23, 31]. However, PAs are frequently established in remote regions facing less pressure than regions with higher human presence, which makes their assessment challenging [32]. This has led to the use of counterfactual approaches to compare before and after the intervention and/or comparing PAs with non-protected land [28, 33, 34]. For example, Ahmadia et al. [35] conducted a study using statistical matching to evaluate the effectiveness of marine protected areas (MPAs) in conserving fish populations in Indonesia. Remote sensing techniques have also been used to compare deforestation rates over time between protected and unprotected areas. Demonstrating that deforestation rates are significantly lower in PAs [36, 37]. However, large-scale evaluations of the effectiveness of PAs in addressing specific threats such as overexploitation and invasive species are lacking [18].

To evaluate management strategies, different criteria, indicators, and methodologies have arisen to evaluate the effectiveness of PAs [17, 33, 38], leading to more than 50 different tools to assess management effectiveness [11, 33, 39]. Protected Areas Management Effectiveness (PAME) evaluations aim to assess how well PAs are being managed, especially on which management strategies successfully preserve values and achieve goals and objectives [40]. Self-assessment surveys are typically used to measure progress toward management goals. In some cases, and when available, quantitative data is also incorporated to provide a more objective estimation of effectiveness [41], nonetheless, the evaluation remains subjective. Most methodologies are based on the IUCN WCPA framework for PAME, which seeks to assess how PAs are being managed by covering three main themes: design matters referring to the individual sites and the PA networks, capability and suitability of management strategies, and achievement of PA objectives and biodiversity conservation values [40]. For example, the Management Effectiveness Tracking Tool (METT) questionnaire-based approach is currently the most widely used PAME tool worldwide [41]. The latest version METT-4 places greater emphasis on the threat assessment section, expanding it to include detailed information on threat extension, severity, and management response. METT-4 also includes sections for gathering key information on the PAs attributes and a questionnaire consisting of 38 questions to evaluate management elements [42].

Overall, most studies on the effectiveness of PAs have focused on ecological features while paying less attention to their potential to reduce threats, which are one of the main drivers of biodiversity loss [26, 43, 44]. Few approaches have focused directly on the impact of threat mitigation strategies, such as the Threat Reduction Assessment (TRA). TRA quantifies the effectiveness of conservation actions that have been implemented. This method calculates an index that summarizes the percentage of effectiveness of the project in reducing the magnitude of priority threats [45, 46]. PAs have been established in response to threats to biodiversity, but these threats are also the catalyst for creating management strategies for PAs. By analyzing the impact of threat reduction schemes, we will also better understand a relevant component of the management effectiveness of PAs.

This systematic review will analyze relevant literature that has studied the role of PAs in addressing threats to biodiversity. We aim to identify whether PAs have reduced threats and what characteristics have helped or hindered their effectiveness. While a previous systematic review has addressed how PAs respond to the specific threat of forest loss [23], our review will examine the efficiency of PAs in reducing all anthropogenic threats to biodiversity.

We will use the definition of threats proposed by [4] in studies assessing direct threats at any spatial scope (local, national, regional, continental, or global). The IUCN Threats Classification Scheme (Version 3.3) will be used to reference direct threats. The IUCN Threats Classification Scheme is widely recognized and used as a comprehensive framework for assessing and classifying threats to species and ecosystems. It provides a standardized approach to identifying and describing threats that directly impact biodiversity. Using this scheme, we aim to ensure consistency and comparability in assessing direct threats across different PAs. Our study focuses explicitly on direct threats. These threats impact biodiversity and are often caused by human activities, such as habitat destruction, pollution, overexploitation, invasive species, and climate change. Studies that have considered proxies of threats, such as the Human Footprint, Human Pressure, and other indices that have been developed to measure human pressure, will also be included in the research. This study will incorporate all designations of PAs in which the main purpose seeks to conserve biodiversity. We will include studies from all realms: terrestrial, marine, and freshwater.

### Stakeholder engagement

While the objective and questions of this study have arisen from the authors' scientific motivation, stakeholder engagement is crucial for effective conservation. Therefore, we acknowledge the need for stakeholder involvement in future research. Nonetheless, we expect that the study findings will significantly impact conservationists, protected area managers, and decision-makers. By assessing the success of current management strategies and methods for addressing changes in threats to biodiversity, this research will improve our understanding of the effectiveness of PAs in addressing threats. Furthermore, the information gathered can serve as evidence to formulate regulations related to Protected Area Management and Evaluation (PAME) and identify PAs' main strengths and weaknesses in facing threats based on their geographical location and socio-economic-ecological characteristics. In addition, this review will provide significant findings for national decision-making.

It will offer insights from various regions worldwide on what strategies and approaches work best in threat abatement within PAs. This knowledge transfer is particularly crucial for ecosystems facing similar problems in threat abatement. By identifying the factors contributing to PAs' success in mitigating threats to biodiversity, targeted policies and management strategies can be developed to promote conservation success and contribute to the Global Biodiversity Framework. Therefore, this study is an essential step toward achieving effective conservation management and achieving the goals of the Global Biodiversity Framework.

## Objective of the review

This systematic review aims to identify peer-reviewed and grey literature studies investigating how effective PAs are for reducing threats to biodiversity. Emphasis will be on studies investigating changes in threats over time, how these changes relate to PAs, and the protection and intervention strategies implemented in the PAs. The primary question is: How effective are PAs for reducing threats to biodiversity? The study question components are outlined in Table 1. In addition, the following number of secondary questions are used to add precision to the facets of the primary question:

- What threats are being studied?
- How are threats being assessed? What type of study designs have been used?
- What is the state of the evidence: number of studies, study location, intervention type, type of threats, and type of PAs (If available)?
- What actions have been implemented to reduce threats in PAs, and what evidence exists of their effectiveness?
- Is the relationship between threats and biodiversity considered?
- What factors are associated with the success or failure of threats reduction over time?

**Table 1:** Components of the review question

Population	Intervention	Comparator	Outcome
Areas experiencing threats to biodiversity	Establishing protected and conserved areas	No protection or before establishment	Difference in threat state

## Methods

This systematic review protocol follows the Collaboration for Environmental Evidence (CEE) guidelines and complies with the ROSES reporting standards, which are included as an Additional file 1.

### Searching for articles

For this phase of the study, we will consider peer-reviewed scientific articles (research articles and reviews) and grey literature. The search will not be restricted in terms of date of publication. The review will focus on English-language publications due to limited resources for working with other languages, especially during screening, full review, quality appraisal, and data extraction.

#### Search terms, strings and publication databases

To identify search terms for the Population, Intervention, Comparator, Outcome (PICO) categories, we thoroughly evaluated related terms based on consensus among the authors, in line with our research question and goals (Table 2). Our study aims to investigate the impact of PAs on addressing threats to biodiversity and evaluate the outcomes of PAs. For maximum coverage of relevant literature and a broad range of reported threats, we deliberately chose search terms that are broad in scope. Therefore, we employed broad search terms to cover a wide range of PA types and threats, without limiting the search terms to specific IUCN PA typologies or threat terms such as "land use change," "agriculture," "invasive species," or "deforestation." This approach was taken to prevent any potential bias in the results, especially considering that the IUCN threat classification scheme lists over 117 direct threats. The keywords in each category will be combined using the Boolean operator ‘OR’; then, the three categories will be combined using ‘AND’. Additionally, an asterisk (*) is a ‘wildcard’ that represents any group of characters, including no character, while a dollar sign ($) represents zero or one character.

**Table 2:** Key words for literature search

Categories	Key words
P: Areas experiencing threats to biodiversity	Threat, human impact, human pressure, anthropogenic impact, human activity, stressor, anthropogenic pressure
I: Protected areas	Protected areas, conservation areas, nature reserve, sanctuary, national park, biosphere reserve, biodiversity reserve, wildlife habitat
O: Difference in threat state	Reduce, effectiveness, impact

The PICO-related keywords will be used in search strings to query databases and search engines. The proposed search string was selected for use in the Web of Science—Core Collection (search by topic) and Scopus (Search by TITLE-ABS-KEY), using the credential of the Copenhagen University library and the number of publications available by the 14th of August of 2023 (Table 3) (see Additional file 2).

**Table 3:** Search string that will be used in the search of the literature in the Web of Science Core Collection and Scopus

Search string	
Threats to biodiversity	threat$ OR "human impact*" OR "human pressure*" OR” anthropogenic impact*" OR "human activity" OR stressor OR “anthropogenic pressure” AND
Measure the change of threats	reduc* OR effective* OR impact AND
Protected areas	"protected area*" OR "conserv* area" OR "nature reserve" OR sanctuar* OR "national park*" OR "biosphere reserve*" OR "biodiversity reserve*" OR "wildlife habitat*"

After testing the search strings, we applied the following filtering criteria: Filter I (WoS and Scopus): Filtered on the website by language “English”, document type as “Article” and “Review”. Additional filter by subject was performed in WoS (I): “Environmental Sciences”, “Biodiversity Conservation” “Multidisciplinary Sciences”, and Scopus: “Environmental Sciences”.

#### Search comprehensiveness

Benchmark studies were selected based on the expertise of the review team, independent of the search strategy. These studies were chosen for their significant contributions to the subject area, focusing on the role of PAs in addressing threats to biodiversity. Additional studies were identified through separate searches using tools like Google Scholar and artificial intelligence resources, such as "Consensus" (https://consensus.app/search/) and "scite" (https://scite.ai/). In total, 20 highly relevant benchmark papers formed the basis for developing the search strategy and assessing its comprehensiveness. To ensure the adequacy of the search strategy in retrieving pertinent literature, we reviewed the search output for relevant articles, including each benchmark article scoped. We refined the search strategy for articles initially not retrieved by adding keywords until all benchmark articles were successfully captured (Additional file 2: Search comprehensiveness and benchmark studies).

#### Internet and specialist searches

In addition to WoS and Scopus, we will conduct searches in Google and Google Scholar for comprehensive coverage, capturing results not included in the databases and exploring potential sources of grey literature. We will use related terms to the PICO components in our search to identify relevant studies. For the google searches, we will review the initial 50 results and include them if not previously identified. In addition, we will search on the websites of organizations that have developed or collected reports related to the topic and have the option to search by keywords in the database. We will manually select reports and studies related to threat assessments in PAs to ensure a thorough search. If keyword searching (e.g., threats and protected areas) is not available, we will communicate once with the organizations if it is known that they have assessed threats in PAs. Grey literature will be sought from various sources, including the Wildlife Conservation Society (WCS) International Union for Nature Conservation (IUCN) and the Global Environmental Facility (GEF). Furthermore, we will explore regional or international conservation networks and partnerships, such as the World Wide Fund for Nature (WWF), Conservation International, BirdLife International, and regional sections of the Society for Conservation Biology (SCB). These organizations frequently publish reports, working papers, and case studies pertaining to PAs.

### Article screening and study eligibility criteria

#### Screening process

The compiled library will undergo meticulous duplicate removal using EndNote 20 [47] and 'Covidence'. Covidence is a web-based collaboration software platform that streamlines the production of systematic reviews, will be used to facilitate the duplicate removal and the screening process (https://www.covidence.org/home). Furthermore, any remaining duplicates will be examined using the "*find_duplicates*" function from the R package 'revtools' [48]. A preliminary trial of the deduplication procedure has been done. Out of the total 7825 articles (Additional file 2), EndNote found 6290 unique studies. Subsequently, employing Covidence facilitated the removal of an additional 607 duplicate entries. Following the deduplication removal, a thorough evaluation of titles and abstracts will be undertaken, guided by the provided eligibility criteria. Items with uncertain eligibility will be preserved for subsequent analysis.

The authors and the review team (additional potential collaborators involving volunteers and future authors) will divide the screening into two steps. Firstly, a title and abstract level screening and, secondly, at the full-text level. To support this process, we will use R package 'revtools' “*read_bibliography”* for the abstract screening phase and Covidence for the full-text review.

As a check for consistency at the title and abstract stage, the review team will assess a random subset of 10% of the total articles found. For this subset, we will test agreement using the Kappa index and define the threshold of $Kappa\ge 0.6$ as a moderate agreement. All discrepancies will be discussed and reviewed to increase consistency and if necessary, increase the specificity of the inclusion and exclusion criteria. In cases of uncertainty, we will tend towards inclusion; thus, articles will be passed on to the second step and assessed at the full-text level. Then, each article found to be potentially eligible based on the abstract will be evaluated for inclusion by reviewers studying the full text. To maintain consistency in our review process, at least two team members will independently assess 10% of the articles selected at the abstract level and discuss disagreements. This sample will be used to evaluate the inclusion or exclusion of articles during the full-text screening stage. During the full-text screening stage, we will document the excluded studies and the supporting reasons for their exclusion. Moreover, if a review team member is an author(s) of the studies to be considered, they will have no role in decisions regarding inclusion or critical appraisal, and other reviewers will do this instead.

#### Eligibility criteria

According to the PICO components, the selection of the inclusion criteria is based on identifying studies that investigate the effectiveness of PAs in controlling threats to biodiversity and contribute to our understanding of the role of PAs in biodiversity conservation. Specifically, we aim to identify studies that measure changes in threats to biodiversity within PAs or their buffer zones (Table 4).

**Table 4:** Inclusion and exclusion criteria for article screening

	Category	Eligibility criteria	Exclusion criteria
Population	Areas experiencing threats to biodiversity	Studies measuring the change of threats state to biodiversity in PAs	The study does not refer to threats to biodiversity or measure threat changes
		The threats reported in the studies must be present in the IUCN threat classification scheme studies. The reported threats must be anthropogenic (no geological events) and can be assessed directly or indirectly, such as proxies of threats measured by human drivers (e.g. Human footprint index)	The study does not refer to human threats. Eg geological events
Intervention	Establishing protected areas	Site(s) designated to conserve biodiversity: including all types and designations of PAs such as national parks, wildlife sanctuaries, nature reserves, biosphere reserves	The study is not focused on specific areas destined for biodiversity conservation as the primary objective
		Worldwide conducted studies	
		All ecological realms and species: terrestrial, freshwater and marine ecosystems	
Comparator	Difference in threat state	Studies measuring changes in threats to PAs	Studies that do not specifically measure the change in threats over time in PAs. e.g. Studies identifying threats without assessing their temporal dynamics
		(a) Temporal Studies: comparing at least two time periods to evaluate the impact of actions, implementation efforts, or similar interventions on the state of threats within PAs	Studies are not focused on measuring threat interventions on PAs. e.g. Studies identifying threats without assessing threats over time
		(b) Comparative Studies: These studies compare the threats inside and outside of PAs or analyze the changes in threats before and after implementing threat control measures over a specific period	
		(c) Management Comparison Studies: These studies examine and compare the outcomes of threats in PAs with different management approaches over time	
Outcome	Measure the change of threats inside PAs	Quantitative evaluation of threats and/or comparison of areas with or without protection, specifically focusing on measuring changes in the state of threats over time	Studies are not referring to threat stateafter an intervention. e.g. Studies measuring changes in species population abundance or other ecological properties instead of threats. e.g. Studies comparing species, population characteristics, abundance, cover, presence, and/or composition among protected and non-protected sites
		Use of direct or indirect measurements to assess changes in the threat state. This includes both direct assessments of threats as well as indirect assessments using proxies or other measurement approaches	
		Studies that collect data through surveys and interviews measure human perception of threat change and transform these experiences into numerical outputs. An example of such an assessment is the Threat Reduction Assessment	
Study type	–	Studies that employ comparison groups and/or utilize before-after (BA) or before-after control-impact (BACI) study designs will be included	Personal views and perspectives, theoretical studies, and models. Studies using observational data with no controls or comparators
Language	–	English	Other languages are excluded

### Study validity assessment

Eligible studies will be critically appraised after the full-text review. The systematic review will utilize the Collaboration for Environmental Evidence (CEE) Critical Appraisal Tool to evaluate the validity and risk of bias of the selected studies [49]. Designed for assessing risk of bias in primary research studies, the tool provides a structured and transparent way of evaluating the quality and relevance of environmental conservation evidence. Three reviewers will independently conduct validity appraisals of the selected studies to maintain consistency. Validity appraisal results for each study and reasons for exclusion will be reported in a separate file.

### Data coding and extraction strategy

An evidence table will be constructed using data extracted from the selected studies, including study characteristics, PA information, and threat assessments. The extracted information will be based on the PICO elements (Table 5). In cases where information is missing, we will declare it as non-reported (–). A minimum of three reviewers will perform the data extraction, and to ensure consistency, a set of ten studies will be first coded together. If uncertainties arise, they will be discussed among reviewers. The methods for data extraction, including additional columns and categories, and synthesis will be refined during the early phases of the review. The data from the selected literature will be extracted and saved in Excel spreadsheets and will be part of the supplementary information of the systematic review. As part of our methods, we pilot-tested the data extraction template on a subset of studies to ensure that it captured all the relevant information and was easy to use. The pilot testing allowed us to refine and adjust the template to ensure it was comprehensive and effective for extracting data from all studies included in the review (Additional file 3: Data extraction).

**Table 5:** Data coding and extraction criteria

Section	Content
Metadata	Authors & Publication year, title, publication type, journal, DOI, scale (e.g., Local, regional, global), ecosystem type, threat assessment method, does the study include a control? What variables are considered? Taxonomic group(s) studied, methods, reported threats, data location within article, comments
Information relating to the inclusion criteria	(a) Population: Reported threats IUCN Threat Classification Scheme V3.3, proxies of threats
	(b) Intervention: PA(s) characteristics: Name (if individual PA reported), number of PAs (When multiple PAs has been assessed, study area or country, year of establishment, management type, additional information
	(c) Comparator: Assessment period, study type, study using control variables? Comparison type of the study (e.g. Time, control variable, management type?)
	(d) Outcome: Threat level assessment after control and/or comparison (Value, effect, and in comparative analysis: Was the PA more effective than the control? effect of PA on overall threats (Positive, neutral, negative), identified factors leading the threat change, additional data (and additional comments
Additional calculations	To be defined according to the findings. Initially, results will be reported as the percentage of effectiveness of the PA/threat control strategies to change the threat state over time (Value and effect tabs) (Positive and negative values refer to positive and negative effects, respectively)

### Potential effect modifiers/reasons for heterogeneity

Effect modifiers leading to heterogeneity in the results will be identified during the full-text screening and recorded for the included studies. Likewise, where applicable, we will collect information on the methods used to assess the impact of potential effect modifiers. Due to the nature of our study, several biogeographic, environmental, and socio-economic factors could result in the heterogeneity of impacts found in different studies. Some of the potential effect modifiers identified in previous studies include the category of PA, governance type, geographical location, and topographic features, size of PA, date and period of establishment, the socioeconomic context of the state or country of PA, ecosystem type, among others [23, 50]. A complete list of effect modifiers will be included in the systematic review.

### Data synthesis and presentation

The data synthesis will comprise an extensive narrative synthesis and a summary of findings using descriptive statistics. The narrative synthesis will describe the strength and validity of the evidence along with the study findings. Tables and figures will be produced to summarise the results and will be available as supplementary information on the systematic review. In the process of data extraction and critical appraisal, steps are taken to minimize bias in the result. Using the categories identified in the critical appraisal, a sensitivity analysis will be conducted to test the effects of the validity assessment (e.g., exclusion of articles) and the robustness of the studied outcomes. While meta-analyses are a powerful tool for synthesizing data, they require a homogeneity of outcomes and methods that might not be present for the included studies of this review due to the heterogeneity of the data and methods used to assess the effect of PAs on threats. However, a meta-analysis will be conducted if the collected data (or a portion thereof) permits meaningful quantitative comparisons.

## Supplementary Information

**Additional file 1**. ROSES form.

**Additional file 2**. Comprehensiveness of the search and list of benchmark studies.

**Additional file 3**. Data extraction test.

## Data Availability

All data generated or analyzed during this study are included in this published article (and its Additional files 1, 2 and 3).
