# Supplementary material for: How effective are protected areas for reducing threats to biodiversity? A systematic review protocol
Source: Environ Evid. 2023 Sep 8;12:18. doi: 10.1186/s13750-023-00311-4 (PMC11378842; doi:10.1186/s13750-023-00311-4)
Supplement: Supplementary file 2 — Additional file 2. Comprehensiveness of the search and list of benchmark studies. [file 13750_2023_311_MOESM2_ESM.docx]

# **Additional file 2 - Assessing the comprehensiveness of the search and benchmark study list**

**Search string 1:**

("protected area$" OR "conserv* area" OR "nature reserve" OR sanctuar* OR "national park*" OR "biosphere reserve*" OR "biodiversity reserve*" OR "wildlife habitat*") **AND**

(threat$ OR "human impact*" OR "human pressure*" OR "anthropogenic impact*" OR "human activit*" OR stressor OR ~~"anthropogenic pressure"~~) **AND**

(impact* OR reduct* ~~OR effective*~~)

**Search string 2:**

("protected area$" OR "conserv* area" OR "nature reserve" OR sanctuar* OR "national park*" OR "biosphere reserve*" OR "biodiversity reserve*" OR "wildlife habitat*") **AND**

(threat$ OR "human impact*" OR "human pressure*" OR "anthropogenic impact*" OR "human activit*" OR stressor OR ~~"anthropogenic pressure"~~) **AND**

(impact* OR reduct* OR effective*)

**Selected search string:**

("protected area$" OR "conserv* area" OR "nature reserve" OR sanctuar* OR "national park*" OR "biosphere reserve*" OR "biodiversity reserve*" OR "wildlife habitat*") **AND**

(threat$ OR "human impact*" OR "human pressure*" OR "anthropogenic impact*" OR "human activit*" OR stressor OR "anthropogenic pressure") **AND**

(impact* OR reduct* OR effective*)

**Specifications of the scoping exercise**

Databases: Web of Science Core Collection & Scopus

Search by: Topic (WoS), Article, title, abstract and keywords (Scopus)

Timespan: All

Document Types: Article and Review

Language: English

Number of results: WoS = 3679 (After filtering)

Scopus = 4146 (After filtering)

Total number of studies (Removing duplicates): 5683

Date of last search: 14-08-2023

Filtering criteria: WoS Environmental sciences, biodiversity conservation, and multidisciplinary sciences. Scopus: Environmental Science

**Testing search string against a test library**

The effectiveness of the search terms in capturing relevant studies was tested by comparing the search results from the scoping exercise against a predefined test library. This test library consisted of 20 studies that were selected as representative examples of the impact of protected areas in addressing threats to biodiversity.

**Assessing search strategy comprehensiveness using benchmark articles**

|  |  | Captured by WoS / Scopus? | | | | |
| --- | --- | --- | --- | --- | --- | --- |
| # | **Reference** | **Search string 1** |  | **Search string 2** |  | **Final search string** |
| 1 | Gong, Minghao, et al. "Measuring the effectiveness of protected area management by comparing habitat utilization and threat dynamics." Biological Conservation 210 (2017): 253-260. | Yes |  | Yes |  | Yes |
| 2 | Feng, Chun‐Ting, et al. "Improving protected area effectiveness through consideration of different human‐pressure baselines." Conservation Biology 36.4 (2022): e13887. | Yes |  | Yes |  | Yes |
| 3 | Matar, Diane A., and Brandon P. Anthony. "Application of modified threat reduction assessments in Lebanon." Conservation Biology 24.5 (2010): 1174-1181. | Yes |  | Yes |  | Yes |
| 4 | Milatović, Luna, Brandon P. Anthony, and Anthony Swemmer. "Estimating conservation effectiveness across protected areas in Limpopo Province, South Africa." Koedoe: African Protected Area Conservation and Science 61.1 (2019): 1-10. | Yes |  | Yes |  | Yes |
| 5 | Mugisha, Arthur R., and Susan K. Jacobson. "Threat reduction assessment of conventional and community-based conservation approaches to managing protected areas in Uganda." Environmental conservation 31.3 (2004): 233-241. | Yes |  | Yes |  | Yes |
| 6 | Tan, Linyi, Guancheng Guo, and Shicheng Li. "The Sanjiangyuan Nature Reserve is partially effective in mitigating human pressures." Land 11.1 (2021): 43. | **No** |  | Yes |  | Yes |
| 7 | Mucova, Serafino Afonso Rui, et al. "Assessment of land use and land cover changes from 1979 to 2017 and biodiversity & land management approach in Quirimbas National Park, Northern Mozambique, Africa." Global ecology and conservation 16 (2018): e00447. | Yes |  | Yes |  | Yes |
| 8 | Shah, Payal, et al. "What determines the effectiveness of national protected area networks?." Environmental Research Letters 16.7 (2021): 074017. | Yes |  | Yes |  | Yes |
| 9 | Geldmann, Jonas, et al. "A global-level assessment of the effectiveness of protected areas at resisting anthropogenic pressures." Proceedings of the National Academy of Sciences 116.46 (2019): 23209-23215. | Yes |  | Yes |  | Yes |
| 10 | Athira, K., et al. "Habitat monitoring and conservation prioritisation of protected areas in Western Ghats, Kerala, India." Environmental Monitoring and Assessment 189 (2017): 1-13. | Yes |  | Yes |  | Yes |
| 11 | Andrade-Núñez, María José, and T. Mitchell Aide. "Using nighttime lights to assess infrastructure expansion within and around protected areas in South America." Environmental Research Communications 2.2 (2020): 021002. | Yes |  | Yes |  | Yes |
| 12 | Graham, Victoria, et al. "Southeast Asian protected areas are effective in conserving forest cover and forest carbon stocks compared to unprotected areas." Scientific reports 11.1 (2021): 23760. | **No** |  | Yes |  | Yes |
| 13 | Zupan, Mirta, et al. "How good is your marine protected area at curbing threats?." Biological Conservation 221 (2018): 237-245. | Yes |  | Yes |  | Yes |
| 14 | Vačkář, David, et al. "Human transformation of ecosystems: comparing protected and unprotected areas with natural baselines." Ecological indicators 66 (2016): 321-328. | **No** |  | **No** |  | Yes |
| 15 | Ye, Xin, et al. "Assessing local and surrounding threats to the protected area network in a biodiversity hotspot: the Hengduan Mountains of Southwest China." PloS one 10.9 (2015): e0138533. | **No** |  | Yes |  | Yes |
| 16 | Anderson, Emily, and Christos Mammides. "The role of protected areas in mitigating human impact in the world’s last wilderness areas." Ambio 49.2 (2020): 434-441. | Yes |  | Yes |  | Yes |
| 17 | Gallardo, Belinda, et al. "Protected areas offer refuge from invasive species spreading under climate change." Global change biology 23.12 (2017): 5331-5343. | Yes |  | Yes |  | Yes |
| 18 | Carranza, Tharsila, et al. "Protected area effectiveness in reducing conversion in a rapidly vanishing ecosystem: the Brazilian Cerrado." Conservation Letters 7.3 (2014): 216-223. | **No** |  | Yes |  | Yes |
| 19 | Moore, Jennifer F., et al. "Are ranger patrols effective in reducing poaching‐related threats within protected areas?." Journal of Applied Ecology 55.1 (2018): 99-107. | **No** |  | Yes |  | Yes |
| 20 | Brown, Kerry A., et al. "Protected area safeguard tree and shrub communities from degradation and invasion: a case study in eastern Madagascar." Environmental Management 44 (2009): 136-148. | Yes |  | Yes |  | Yes |
| % of papers captured by search string: | | 70% |  | 95% |  | 100% |
